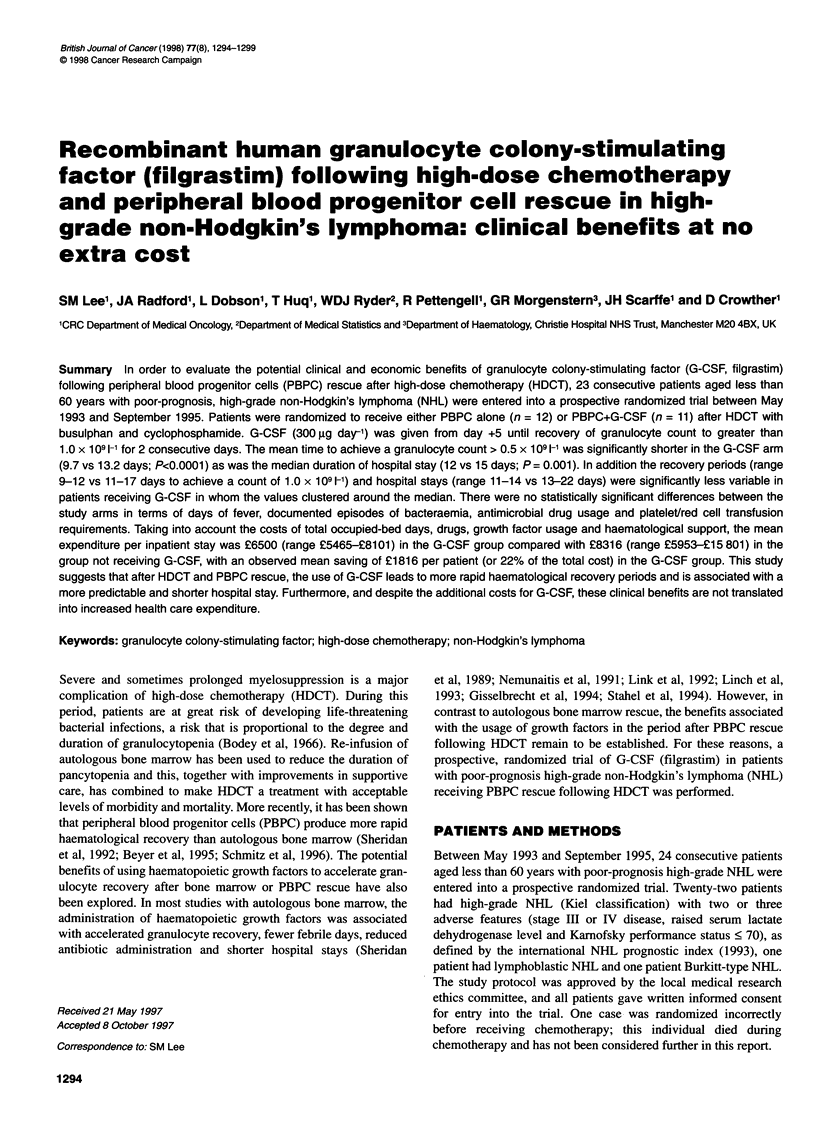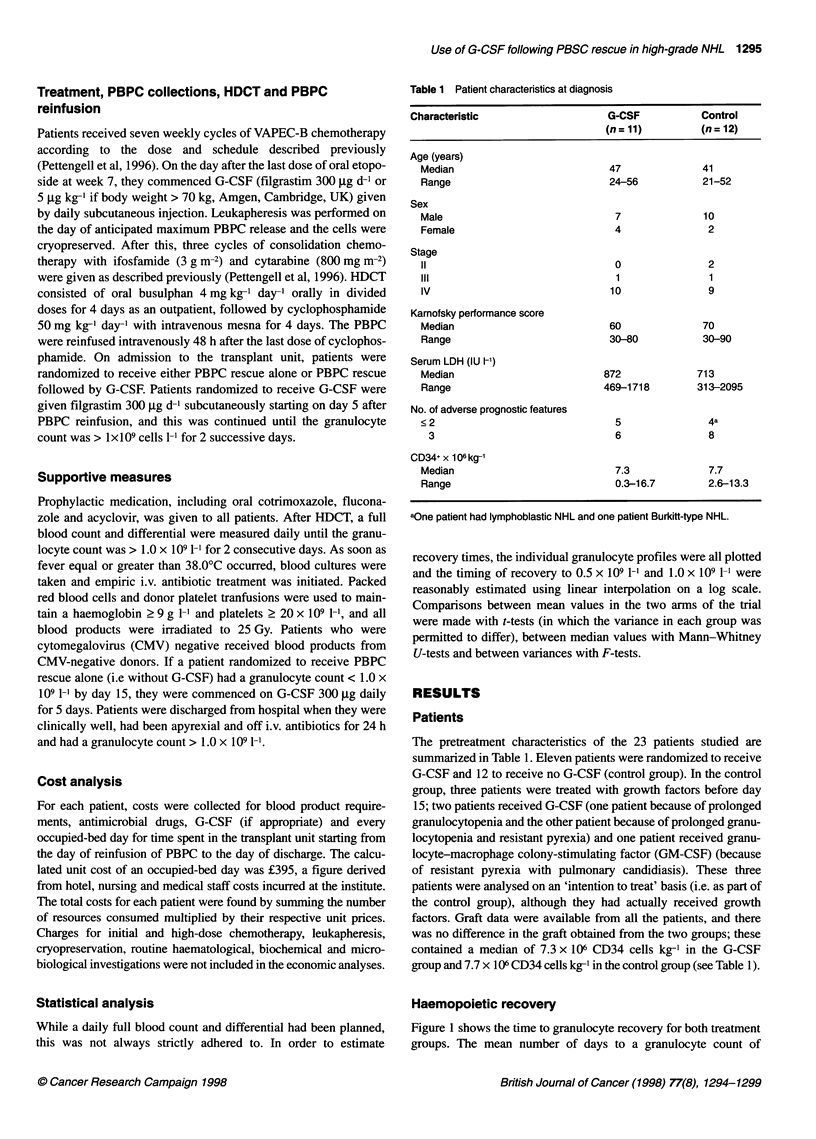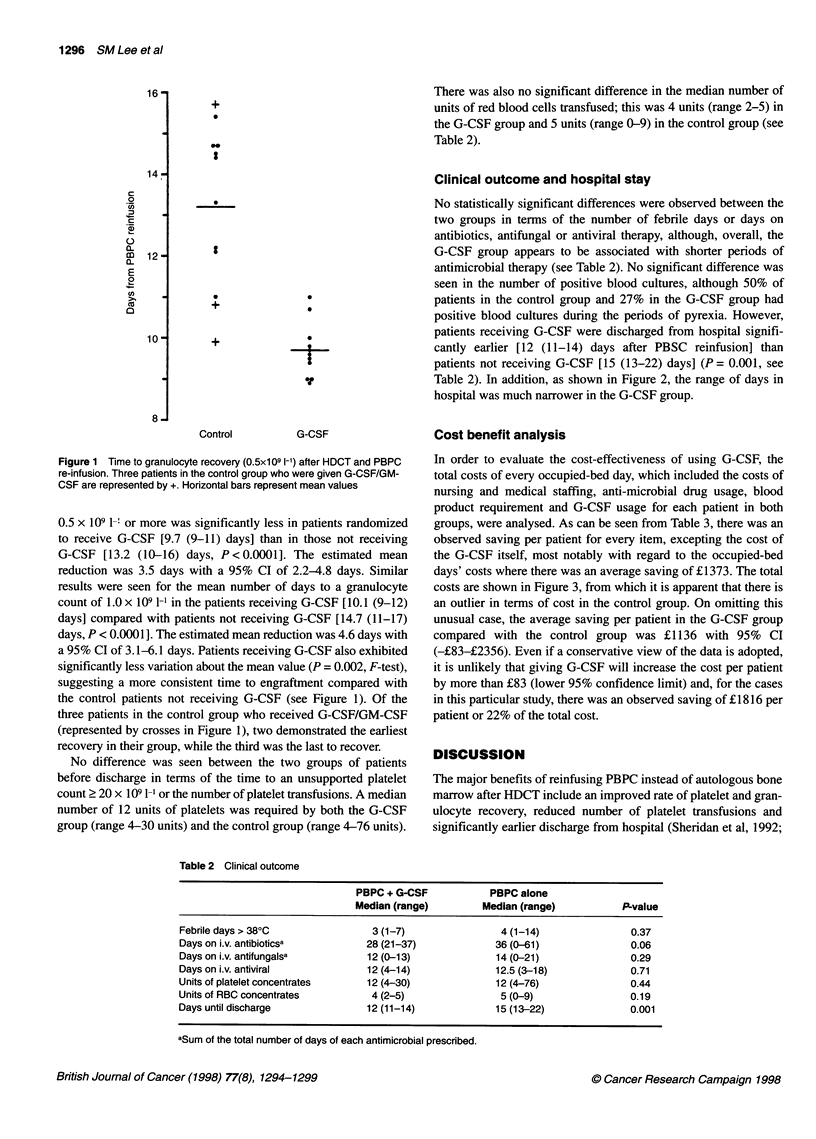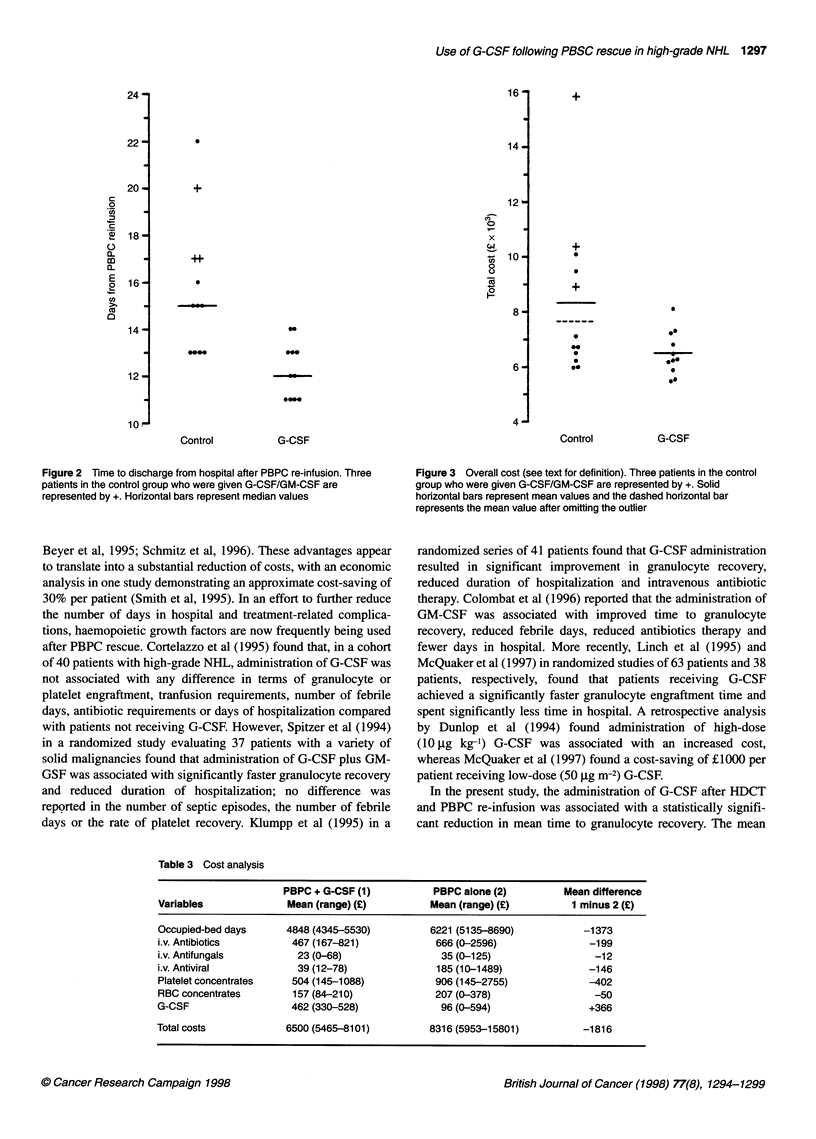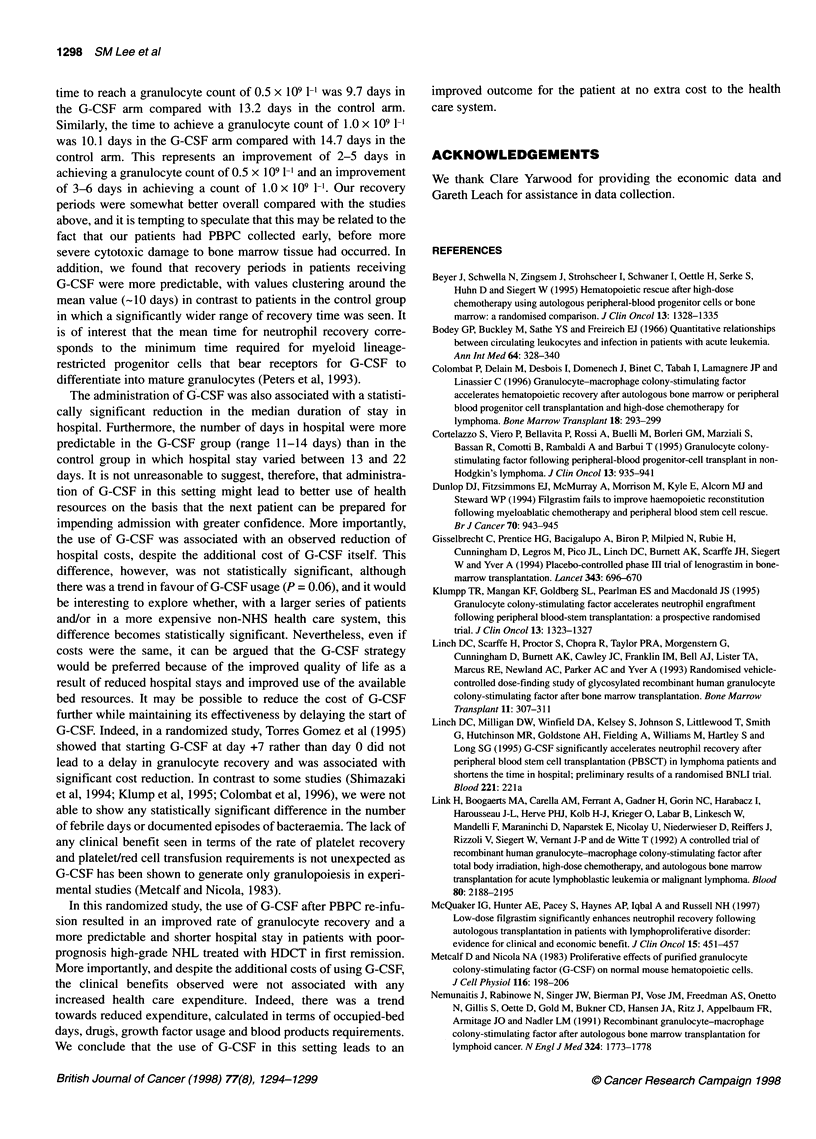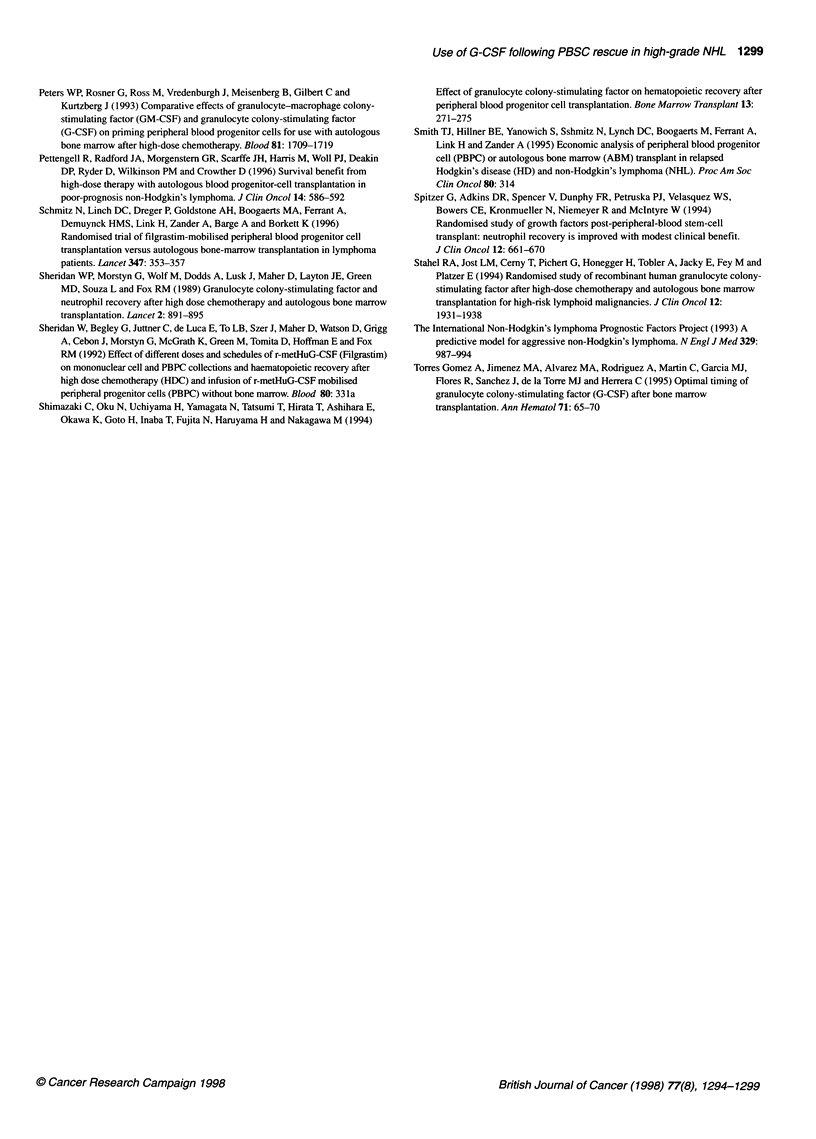# Recombinant human granulocyte colony-stimulating factor (filgrastim) following high-dose chemotherapy and peripheral blood progenitor cell rescue in high-grade non-Hodgkin's lymphoma: clinical benefits at no extra cost.

**DOI:** 10.1038/bjc.1998.216

**Published:** 1998-04

**Authors:** S. M. Lee, J. A. Radford, L. Dobson, T. Huq, W. D. Ryder, R. Pettengell, G. R. Morgenstern, J. H. Scarffe, D. Crowther

**Affiliations:** CRC Department of Medical Oncology, Christie Hospital NHS Trust, Manchester, UK.

## Abstract

In order to evaluate the potential clinical and economic benefits of granulocyte colony-stimulating factor (G-CSF, filgrastim) following peripheral blood progenitor cells (PBPC) rescue after high-dose chemotherapy (HDCT), 23 consecutive patients aged less than 60 years with poor-prognosis, high-grade non-Hodgkin's lymphoma (NHL) were entered into a prospective randomized trial between May 1993 and September 1995. Patients were randomized to receive either PBPC alone (n = 12) or PBPC+G-CSF (n = 11) after HDCT with busulphan and cyclophosphamide. G-CSF (300 microg day[-1]) was given from day +5 until recovery of granulocyte count to greater than 1.0 x 10(9) l(-1) for 2 consecutive days. The mean time to achieve a granulocyte count > 0.5 x 10(9) l(-1) was significantly shorter in the G-CSF arm (9.7 vs 13.2 days; P<0.0001) as was the median duration of hospital stay (12 vs 15 days; P = 0.001). In addition the recovery periods (range 9-12 vs 11-17 days to achieve a count of 1.0 x 10(9) l[-1]) and hospital stays (range 11-14 vs 13-22 days) were significantly less variable in patients receiving G-CSF in whom the values clustered around the median. There were no statistically significant differences between the study arms in terms of days of fever, documented episodes of bacteraemia, antimicrobial drug usage and platelet/red cell transfusion requirements. Taking into account the costs of total occupied-bed days, drugs, growth factor usage and haematological support, the mean expenditure per inpatient stay was pound sterling 6500 (range pound sterling 5465-pound sterling 8101) in the G-CSF group compared with pound sterling 8316 (range pound sterling 5953-pound sterling 15,801) in the group not receiving G-CSF, with an observed mean saving of 1816 per patient (or 22% of the total cost) in the G-CSF group. This study suggests that after HDCT and PBPC rescue, the use of G-CSF leads to more rapid haematological recovery periods and is associated with a more predictable and shorter hospital stay. Furthermore, and despite the additional costs for G-CSF, these clinical benefits are not translated into increased health care expenditure.


*British Journal of Cancer* (1998) **77**(8), 1294–1299
© 1998 Cancer Research Campaign

### Recombinant human granulocyte colony-stimulating factor (filgrastim) following high-dose chemotherapy and peripheral blood progenitor cell rescue in high-grade non-Hodgkin's lymphoma: clinical benefits at no extra cost

**SM Lee[1], JA Radford[1], L Dobson[1], T Huq[1], WDJ Ryder[2], R Pettengell[1], GR Morgenstern[3], JH Scarffe[1] and D Crowther[1]**

[1]CRC Department of Medical Oncology, [2]Department of Medical Statistics and [3]Department of Haematology, Christie Hospital NHS Trust, Manchester M20 4BX, UK

**Summary** In order to evaluate the potential clinical and economic benefits of granulocyte colony-stimulating factor (G-CSF, filgrastim) following peripheral blood progenitor cells (PBPC) rescue after high-dose chemotherapy (HDCT), 23 consecutive patients aged less than 60 years with poor-prognosis, high-grade non-Hodgkin's lymphoma (NHL) were entered into a prospective randomized trial between May 1993 and September 1995. Patients were randomized to receive either PBPC alone ($n$ = 12) or PBPC+G-CSF ($n$ = 11) after HDCT with busulphan and cyclophosphamide. G-CSF (300 $\mu$g day$^{-1}$) was given from day +5 until recovery of granulocyte count to greater than $1.0 \times 10^9$ l$^{-1}$ for 2 consecutive days. The mean time to achieve a granulocyte count > $0.5 \times 10^9$ l$^{-1}$ was significantly shorter in the G-CSF arm (9.7 vs 13.2 days; $P$<0.0001) as was the median duration of hospital stay (12 vs 15 days; $P$ = 0.001). In addition the recovery periods (range 9–12 vs 11–17 days to achieve a count of $1.0 \times 10^9$ l$^{-1}$) and hospital stays (range 11–14 vs 13–22 days) were significantly less variable in patients receiving G-CSF in whom the values clustered around the median. There were no statistically significant differences between the study arms in terms of days of fever, documented episodes of bacteraemia, antimicrobial drug usage and platelet/red cell transfusion requirements. Taking into account the costs of total occupied-bed days, drugs, growth factor usage and haematological support, the mean expenditure per inpatient stay was £6500 (range £5465–£8101) in the G-CSF group compared with £8316 (range £5953–£15 801) in the group not receiving G-CSF, with an observed mean saving of £1816 per patient (or 22% of the total cost) in the G-CSF group. This study suggests that after HDCT and PBPC rescue, the use of G-CSF leads to more rapid haematological recovery periods and is associated with a more predictable and shorter hospital stay. Furthermore, and despite the additional costs for G-CSF, these clinical benefits are not translated into increased health care expenditure.

**Keywords:** granulocyte colony-stimulating factor; high-dose chemotherapy; non-Hodgkin's lymphoma

Severe and sometimes prolonged myelosuppression is a major complication of high-dose chemotherapy (HDCT). During this period, patients are at great risk of developing life-threatening bacterial infections, a risk that is proportional to the degree and duration of granulocytopenia (Bodey et al, 1966). Re-infusion of autologous bone marrow has been used to reduce the duration of pancytopenia and this, together with improvements in supportive care, has combined to make HDCT a treatment with acceptable levels of morbidity and mortality. More recently, it has been shown that peripheral blood progenitor cells (PBPC) produce more rapid haematological recovery than autologous bone marrow (Sheridan et al, 1992; Beyer et al, 1995; Schmitz et al, 1996). The potential benefits of using haematopoietic growth factors to accelerate granulocyte recovery after bone marrow or PBPC rescue have also been explored. In most studies with autologous bone marrow, the administration of haematopoietic growth factors was associated with accelerated granulocyte recovery, fewer febrile days, reduced antibiotic administration and shorter hospital stays (Sheridan et al, 1989; Nemunaitis et al, 1991; Link et al, 1992; Linch et al, 1993; Gisselbrecht et al, 1994; Stahel et al, 1994). However, in contrast to autologous bone marrow rescue, the benefits associated with the usage of growth factors in the period after PBPC rescue following HDCT remain to be established. For these reasons, a prospective, randomized trial of G-CSF (filgrastim) in patients with poor-prognosis high-grade non-Hodgkin's lymphoma (NHL) receiving PBPC rescue following HDCT was performed.

*Received 21 May 1997*
*Accepted 8 October 1997*

*Correspondence to:* SM Lee

### PATIENTS AND METHODS

Between May 1993 and September 1995, 24 consecutive patients aged less than 60 years with poor-prognosis high-grade NHL were entered into a prospective randomized trial. Twenty-two patients had high-grade NHL (Kiel classification) with two or three adverse features (stage III or IV disease, raised serum lactate dehydrogenase level and Karnofsky performance status ≤ 70), as defined by the international NHL prognostic index (1993), one patient had lymphoblastic NHL and one patient Burkitt-type NHL. The study protocol was approved by the local medical research ethics committee, and all patients gave written informed consent for entry into the trial. One case was randomized incorrectly before receiving chemotherapy; this individual died during chemotherapy and has not been considered further in this report.

### Treatment, PBPC collections, HDCT and PBPC reinfusion

Patients received seven weekly cycles of VAPEC-B chemotherapy according to the dose and schedule described previously (Pettengell et al, 1996). On the day after the last dose of oral etoposide at week 7, they commenced G-CSF (filgrastim 300 μg $d^{-1}$ or 5 μg $kg^{-1}$ if body weight > 70 kg, Amgen, Cambridge, UK) given by daily subcutaneous injection. Leukapheresis was performed on the day of anticipated maximum PBPC release and the cells were cryopreserved. After this, three cycles of consolidation chemotherapy with ifosfamide (3 g $m^{-2}$) and cytarabine (800 mg $m^{-2}$) were given as described previously (Pettengell et al, 1996). HDCT consisted of oral busulphan 4 mg $kg^{-1}$ $day^{-1}$ orally in divided doses for 4 days as an outpatient, followed by cyclophosphamide 50 mg $kg^{-1}$ $day^{-1}$ with intravenous mesna for 4 days. The PBPC were reinfused intravenously 48 h after the last dose of cyclophosphamide. On admission to the transplant unit, patients were randomized to receive either PBPC rescue alone or PBPC rescue followed by G-CSF. Patients randomized to receive G-CSF were given filgrastim 300 μg $d^{-1}$ subcutaneously starting on day 5 after PBPC reinfusion, and this was continued until the granulocyte count was > $1 \times 10^9$ cells $l^{-1}$ for 2 successive days.

### Supportive measures

Prophylactic medication, including oral cotrimoxazole, fluconazole and acyclovir, was given to all patients. After HDCT, a full blood count and differential were measured daily until the granulocyte count was > $1.0 \times 10^9$ $l^{-1}$ for 2 consecutive days. As soon as fever equal or greater than 38.0°C occurred, blood cultures were taken and empiric i.v. antibiotic treatment was initiated. Packed red blood cells and donor platelet tranfusions were used to maintain a haemoglobin ≥ 9 g $l^{-1}$ and platelets ≥ $20 \times 10^9$ $l^{-1}$, and all blood products were irradiated to 25 Gy. Patients who were cytomegalovirus (CMV) negative received blood products from CMV-negative donors. If a patient randomized to receive PBPC rescue alone (i.e without G-CSF) had a granulocyte count < $1.0 \times 10^9$ $l^{-1}$ by day 15, they were commenced on G-CSF 300 μg daily for 5 days. Patients were discharged from hospital when they were clinically well, had been apyrexial and off i.v. antibiotics for 24 h and had a granulocyte count > $1.0 \times 10^9$ $l^{-1}$.

### Cost analysis

For each patient, costs were collected for blood product requirements, antimicrobial drugs, G-CSF (if appropriate) and every occupied-bed day for time spent in the transplant unit starting from the day of reinfusion of PBPC to the day of discharge. The calculated unit cost of an occupied-bed day was £395, a figure derived from hotel, nursing and medical staff costs incurred at the institute. The total costs for each patient were found by summing the number of resources consumed multiplied by their respective unit prices. Charges for initial and high-dose chemotherapy, leukapheresis, cryopreservation, routine haematological, biochemical and microbiological investigations were not included in the economic analyses.

### Statistical analysis

While a daily full blood count and differential had been planned, this was not always strictly adhered to. In order to estimate recovery times, the individual granulocyte profiles were all plotted and the timing of recovery to $0.5 \times 10^9$ $l^{-1}$ and $1.0 \times 10^9$ $l^{-1}$ were reasonably estimated using linear interpolation on a log scale. Comparisons between mean values in the two arms of the trial were made with *t*-tests (in which the variance in each group was permitted to differ), between median values with Mann–Whitney *U*-tests and between variances with *F*-tests.

**Table 1** Patient characteristics at diagnosis

| Characteristic | G-CSF ($n$ = 11) | Control ($n$ = 12) |
|---|---|---|
| Age (years) | | |
| Median | 47 | 41 |
| Range | 24–56 | 21–52 |
| Sex | | |
| Male | 7 | 10 |
| Female | 4 | 2 |
| Stage | | |
| II | 0 | 2 |
| III | 1 | 1 |
| IV | 10 | 9 |
| Karnofsky performance score | | |
| Median | 60 | 70 |
| Range | 30–80 | 30–90 |
| Serum LDH (IU $l^{-1}$) | | |
| Median | 872 | 713 |
| Range | 469–1718 | 313–2095 |
| No. of adverse prognostic features | | |
| ≤ 2 | 5 | 4[a] |
| 3 | 6 | 8 |
| $CD34^+ \times 10^6$ $kg^{-1}$ | | |
| Median | 7.3 | 7.7 |
| Range | 0.3–16.7 | 2.6–13.3 |

[a]One patient had lymphoblastic NHL and one patient Burkitt-type NHL.

### RESULTS

### Patients

The pretreatment characteristics of the 23 patients studied are summarized in Table 1. Eleven patients were randomized to receive G-CSF and 12 to receive no G-CSF (control group). In the control group, three patients were treated with growth factors before day 15; two patients received G-CSF (one patient because of prolonged granulocytopenia and the other patient because of prolonged granulocytopenia and resistant pyrexia) and one patient received granulocyte–macrophage colony-stimulating factor (GM-CSF) (because of resistant pyrexia with pulmonary candidiasis). These three patients were analysed on an 'intention to treat' basis (i.e. as part of the control group), although they had actually received growth factors. Graft data were available from all the patients, and there was no difference in the graft obtained from the two groups; these contained a median of $7.3 \times 10^6$ CD34 cells $kg^{-1}$ in the G-CSF group and $7.7 \times 10^6$ CD34 cells $kg^{-1}$ in the control group (see Table 1).

### Haemopoietic recovery

Figure 1 shows the time to granulocyte recovery for both treatment groups. The mean number of days to a granulocyte count of

© Cancer Research Campaign 1998

**Figure 1** Time to granulocyte recovery (0.5×$10^9$ $l^{-1}$) after HDCT and PBPC re-infusion. Three patients in the control group who were given G-CSF/GM-CSF are represented by +. Horizontal bars represent mean values

0.5 × $10^9$ $l^{-1}$ or more was significantly less in patients randomized to receive G-CSF [9.7 (9–11) days] than in those not receiving G-CSF [13.2 (10–16) days, $P < 0.0001$]. The estimated mean reduction was 3.5 days with a 95% CI of 2.2–4.8 days. Similar results were seen for the mean number of days to a granulocyte count of 1.0 × $10^9$ $l^{-1}$ in the patients receiving G-CSF [10.1 (9–12) days] compared with patients not receiving G-CSF [14.7 (11–17) days, $P < 0.0001$]. The estimated mean reduction was 4.6 days with a 95% CI of 3.1–6.1 days. Patients receiving G-CSF also exhibited significantly less variation about the mean value ($P = 0.002$, *F*-test), suggesting a more consistent time to engraftment compared with the control patients not receiving G-CSF (see Figure 1). Of the three patients in the control group who received G-CSF/GM-CSF (represented by crosses in Figure 1), two demonstrated the earliest recovery in their group, while the third was the last to recover.

No difference was seen between the two groups of patients before discharge in terms of the time to an unsupported platelet count ≥ 20 × $10^9$ $l^{-1}$ or the number of platelet transfusions. A median number of 12 units of platelets was required by both the G-CSF group (range 4–30 units) and the control group (range 4–76 units). There was also no significant difference in the median number of units of red blood cells transfused; this was 4 units (range 2–5) in the G-CSF group and 5 units (range 0–9) in the control group (see Table 2).

### Clinical outcome and hospital stay

No statistically significant differences were observed between the two groups in terms of the number of febrile days or days on antibiotics, antifungal or antiviral therapy, although, overall, the G-CSF group appears to be associated with shorter periods of antimicrobial therapy (see Table 2). No significant difference was seen in the number of positive blood cultures, although 50% of patients in the control group and 27% in the G-CSF group had positive blood cultures during the periods of pyrexia. However, patients receiving G-CSF were discharged from hospital significantly earlier [12 (11–14) days after PBSC reinfusion] than patients not receiving G-CSF [15 (13–22) days] ($P = 0.001$, see Table 2). In addition, as shown in Figure 2, the range of days in hospital was much narrower in the G-CSF group.

### Cost benefit analysis

In order to evaluate the cost-effectiveness of using G-CSF, the total costs of every occupied-bed day, which included the costs of nursing and medical staffing, anti-microbial drug usage, blood product requirement and G-CSF usage for each patient in both groups, were analysed. As can be seen from Table 3, there was an observed saving per patient for every item, excepting the cost of the G-CSF itself, most notably with regard to the occupied-bed days' costs where there was an average saving of £1373. The total costs are shown in Figure 3, from which it is apparent that there is an outlier in terms of cost in the control group. On omitting this unusual case, the average saving per patient in the G-CSF group compared with the control group was £1136 with 95% CI (–£83–£2356). Even if a conservative view of the data is adopted, it is unlikely that giving G-CSF will increase the cost per patient by more than £83 (lower 95% confidence limit) and, for the cases in this particular study, there was an observed saving of £1816 per patient or 22% of the total cost.

### DISCUSSION

The major benefits of reinfusing PBPC instead of autologous bone marrow after HDCT include an improved rate of platelet and granulocyte recovery, reduced number of platelet transfusions and significantly earlier discharge from hospital (Sheridan et al, 1992;

**Table 2** Clinical outcome

| | PBPC + G-CSF Median (range) | PBPC alone Median (range) | *P*-value |
|---|---|---|---|
| Febrile days > 38°C | 3 (1–7) | 4 (1–14) | 0.37 |
| Days on i.v. antibiotics[a] | 28 (21–37) | 36 (0–61) | 0.06 |
| Days on i.v. antifungals[a] | 12 (0–13) | 14 (0–21) | 0.29 |
| Days on i.v. antiviral | 12 (4–14) | 12.5 (3–18) | 0.71 |
| Units of platelet concentrates | 12 (4–30) | 12 (4–76) | 0.44 |
| Units of RBC concentrates | 4 (2–5) | 5 (0–9) | 0.19 |
| Days until discharge | 12 (11–14) | 15 (13–22) | 0.001 |

[a]Sum of the total number of days of each antimicrobial prescribed.

© Cancer Research Campaign 1998

**Figure 2** Time to discharge from hospital after PBPC re-infusion. Three patients in the control group who were given G-CSF/GM-CSF are represented by +. Horizontal bars represent median values

**Figure 3** Overall cost (see text for definition). Three patients in the control group who were given G-CSF/GM-CSF are represented by +. Solid horizontal bars represent mean values and the dashed horizontal bar represents the mean value after omitting the outlier

Beyer et al, 1995; Schmitz et al, 1996). These advantages appear to translate into a substantial reduction of costs, with an economic analysis in one study demonstrating an approximate cost-saving of 30% per patient (Smith et al, 1995). In an effort to further reduce the number of days in hospital and treatment-related complications, haemopoietic growth factors are now frequently being used after PBPC rescue. Cortelazzo et al (1995) found that, in a cohort of 40 patients with high-grade NHL, administration of G-CSF was not associated with any difference in terms of granulocyte or platelet engraftment, tranfusion requirements, number of febrile days, antibiotic requirements or days of hospitalization compared with patients not receiving G-CSF. However, Spitzer et al (1994) in a randomized study evaluating 37 patients with a variety of solid malignancies found that administration of G-CSF plus GM-GSF was associated with significantly faster granulocyte recovery and reduced duration of hospitalization; no difference was reported in the number of septic episodes, the number of febrile days or the rate of platelet recovery. Klumpp et al (1995) in a randomized series of 41 patients found that G-CSF administration resulted in significant improvement in granulocyte recovery, reduced duration of hospitalization and intravenous antibiotic therapy. Colombat et al (1996) reported that the administration of GM-CSF was associated with improved time to granulocyte recovery, reduced febrile days, reduced antibiotics therapy and fewer days in hospital. More recently, Linch et al (1995) and McQuaker et al (1997) in randomized studies of 63 patients and 38 patients, respectively, found that patients receiving G-CSF achieved a significantly faster granulocyte engraftment time and spent significantly less time in hospital. A retrospective analysis by Dunlop et al (1994) found administration of high-dose (10 $\mu$g kg$^{-1}$) G-CSF was associated with an increased cost, whereas McQuaker et al (1997) found a cost-saving of £1000 per patient receiving low-dose (50 $\mu$g m$^{-2}$) G-CSF.

In the present study, the administration of G-CSF after HDCT and PBPC re-infusion was associated with a statistically significant reduction in mean time to granulocyte recovery. The mean

**Table 3** Cost analysis

| Variables | PBPC + G-CSF (1) Mean (range) (£) | PBPC alone (2) Mean (range) (£) | Mean difference 1 minus 2 (£) |
|---|---|---|---|
| Occupied-bed days | 4848 (4345–5530) | 6221 (5135–8690) | –1373 |
| i.v. Antibiotics | 467 (167–821) | 666 (0–2596) | –199 |
| i.v. Antifungals | 23 (0–68) | 35 (0–125) | –12 |
| i.v. Antiviral | 39 (12–78) | 185 (10–1489) | –146 |
| Platelet concentrates | 504 (145–1088) | 906 (145–2755) | –402 |
| RBC concentrates | 157 (84–210) | 207 (0–378) | –50 |
| G-CSF | 462 (330–528) | 96 (0–594) | +366 |
| Total costs | 6500 (5465–8101) | 8316 (5953–15801) | –1816 |

© Cancer Research Campaign 1998

time to reach a granulocyte count of $0.5 \times 10^9$ $l^{-1}$ was 9.7 days in the G-CSF arm compared with 13.2 days in the control arm. Similarly, the time to achieve a granulocyte count of $1.0 \times 10^9$ $l^{-1}$ was 10.1 days in the G-CSF arm compared with 14.7 days in the control arm. This represents an improvement of 2–5 days in achieving a granulocyte count of $0.5 \times 10^9$ $l^{-1}$ and an improvement of 3–6 days in achieving a count of $1.0 \times 10^9$ $l^{-1}$. Our recovery periods were somewhat better overall compared with the studies above, and it is tempting to speculate that this may be related to the fact that our patients had PBPC collected early, before more severe cytotoxic damage to bone marrow tissue had occurred. In addition, we found that recovery periods in patients receiving G-CSF were more predictable, with values clustering around the mean value (~10 days) in contrast to patients in the control group in which a significantly wider range of recovery time was seen. It is of interest that the mean time for neutrophil recovery corresponds to the minimum time required for myeloid lineage-restricted progenitor cells that bear receptors for G-CSF to differentiate into mature granulocytes (Peters et al, 1993).

The administration of G-CSF was also associated with a statistically significant reduction in the median duration of stay in hospital. Furthermore, the number of days in hospital were more predictable in the G-CSF group (range 11–14 days) than in the control group in which hospital stay varied between 13 and 22 days. It is not unreasonable to suggest, therefore, that administration of G-CSF in this setting might lead to better use of health resources on the basis that the next patient can be prepared for impending admission with greater confidence. More importantly, the use of G-CSF was associated with an observed reduction of hospital costs, despite the additional cost of G-CSF itself. This difference, however, was not statistically significant, although there was a trend in favour of G-CSF usage ($P = 0.06$), and it would be interesting to explore whether, with a larger series of patients and/or in a more expensive non-NHS health care system, this difference becomes statistically significant. Nevertheless, even if costs were the same, it can be argued that the G-CSF strategy would be preferred because of the improved quality of life as a result of reduced hospital stays and improved use of the available bed resources. It may be possible to reduce the cost of G-CSF further while maintaining its effectiveness by delaying the start of G-CSF. Indeed, in a randomized study, Torres Gomez et al (1995) showed that starting G-CSF at day +7 rather than day 0 did not lead to a delay in granulocyte recovery and was associated with significant cost reduction. In contrast to some studies (Shimazaki et al, 1994; Klump et al, 1995; Colombat et al, 1996), we were not able to show any statistically significant difference in the number of febrile days or documented episodes of bacteraemia. The lack of any clinical benefit seen in terms of the rate of platelet recovery and platelet/red cell transfusion requirements is not unexpected as G-CSF has been shown to generate only granulopoiesis in experimental studies (Metcalf and Nicola, 1983).

In this randomized study, the use of G-CSF after PBPC re-infusion resulted in an improved rate of granulocyte recovery and a more predictable and shorter hospital stay in patients with poor-prognosis high-grade NHL treated with HDCT in first remission. More importantly, and despite the additional costs of using G-CSF, the clinical benefits observed were not associated with any increased health care expenditure. Indeed, there was a trend towards reduced expenditure, calculated in terms of occupied-bed days, drugs, growth factor usage and blood products requirements. We conclude that the use of G-CSF in this setting leads to an improved outcome for the patient at no extra cost to the health care system.

### ACKNOWLEDGEMENTS

We thank Clare Yarwood for providing the economic data and Gareth Leach for assistance in data collection.

### REFERENCES

Beyer J, Schwella N, Zingsem J, Strohscheer I, Schwaner I, Oettle H, Serke S, Huhn D and Siegert W (1995) Hematopoietic rescue after high-dose chemotherapy using autologous peripheral-blood progenitor cells or bone marrow: a randomised comparison. *J Clin Oncol* **13**: 1328–1335

Bodey GP, Buckley M, Sathe YS and Freireich EJ (1966) Quantitative relationships between circulating leukocytes and infection in patients with acute leukemia. *Ann Int Med* **64**: 328–340

Colombat P, Delain M, Desbois I, Domenech J, Binet C, Tabah I, Lamagnere JP and Linassier C (1996) Granulocyte–macrophage colony-stimulating factor accelerates hematopoietic recovery after autologous bone marrow or peripheral blood progenitor cell transplantation and high-dose chemotherapy for lymphoma. *Bone Marrow Transplant* **18**: 293–299

Cortelazzo S, Viero P, Bellavita P, Rossi A, Buelli M, Borleri GM, Marziali S, Bassan R, Comotti B, Rambaldi A and Barbui T (1995) Granulocyte colony-stimulating factor following peripheral-blood progenitor-cell transplant in non-Hodgkin's lymphoma. *J Clin Oncol* **13**: 935–941

Dunlop DJ, Fitzsimmons EJ, McMurray A, Morrison M, Kyle E, Alcorn MJ and Steward WP (1994) Filgrastim fails to improve haemopoietic reconstitution following myeloablatic chemotherapy and peripheral blood stem cell rescue. *Br J Cancer* **70**: 943–945

Gisselbrecht C, Prentice HG, Bacigalupo A, Biron P, Milpied N, Rubie H, Cunningham D, Legros M, Pico JL, Linch DC, Burnett AK, Scarffe JH, Siegert W and Yver A (1994) Placebo-controlled phase III trial of lenograstim in bone-marrow transplantation. *Lancet* **343**: 696–670

Klumpp TR, Mangan KF, Goldberg SL, Pearlman ES and Macdonald JS (1995) Granulocyte colony-stimulating factor accelerates neutrophil engraftment following peripheral blood-stem transplantation: a prospective randomised trial. *J Clin Oncol* **13**: 1323–1327

Linch DC, Scarffe H, Proctor S, Chopra R, Taylor PRA, Morgenstern G, Cunningham D, Burnett AK, Cawley JC, Franklin IM, Bell AJ, Lister TA, Marcus RE, Newland AC, Parker AC and Yver A (1993) Randomised vehicle-controlled dose-finding study of glycosylated recombinant human granulocyte colony-stimulating factor after bone marrow transplantation. *Bone Marrow Transplant* **11**: 307–311

Linch DC, Milligan DW, Winfield DA, Kelsey S, Johnson S, Littlewood T, Smith G, Hutchinson MR, Goldstone AH, Fielding A, Williams M, Hartley S and Long SG (1995) G-CSF significantly accelerates neutrophil recovery after peripheral blood stem cell transplantation (PBSCT) in lymphoma patients and shortens the time in hospital; preliminary results of a randomised BNLI trial. *Blood* **221**: 221a

Link H, Boogaerts MA, Carella AM, Ferrant A, Gadner H, Gorin NC, Harabacz I, Harousseau J-L, Herve PHJ, Kolb H-J, Krieger O, Labar B, Linkesch W, Mandelli F, Maraninchi D, Naparstek E, Nicolay U, Niederwieser D, Reiffers J, Rizzoli V, Siegert W, Vernant J-P and de Witte T (1992) A controlled trial of recombinant human granulocyte–macrophage colony-stimulating factor after total body irradiation, high-dose chemotherapy, and autologous bone marrow transplantation for acute lymphoblastic leukemia or malignant lymphoma. *Blood* **80**: 2188–2195

McQuaker IG, Hunter AE, Pacey S, Haynes AP, Iqbal A and Russell NH (1997) Low-dose filgrastim significantly enhances neutrophil recovery following autologous transplantation in patients with lymphoproliferative disorder: evidence for clinical and economic benefit. *J Clin Oncol* **15**: 451–457

Metcalf D and Nicola NA (1983) Proliferative effects of purified granulocyte colony-stimulating factor (G-CSF) on normal mouse hematopoietic cells. *J Cell Physiol* **116**: 198–206

Nemunaitis J, Rabinowe N, Singer JW, Bierman PJ, Vose JM, Freedman AS, Onetto N, Gillis S, Oette D, Gold M, Bukner CD, Hansen JA, Ritz J, Appelbaum FR, Armitage JO and Nadler LM (1991) Recombinant granulocyte–macrophage colony-stimulating factor after autologous bone marrow transplantation for lymphoid cancer. *N Engl J Med* **324**: 1773–1778

© Cancer Research Campaign 1998

Peters WP, Rosner G, Ross M, Vredenburgh J, Meisenberg B, Gilbert C and Kurtzberg J (1993) Comparative effects of granulocyte–macrophage colony-stimulating factor (GM-CSF) and granulocyte colony-stimulating factor (G-CSF) on priming peripheral blood progenitor cells for use with autologous bone marrow after high-dose chemotherapy. *Blood* **81**: 1709–1719

Pettengell R, Radford JA, Morgenstern GR, Scarffe JH, Harris M, Woll PJ, Deakin DP, Ryder D, Wilkinson PM and Crowther D (1996) Survival benefit from high-dose therapy with autologous blood progenitor-cell transplantation in poor-prognosis non-Hodgkin's lymphoma. *J Clin Oncol* **14**: 586–592

Schmitz N, Linch DC, Dreger P, Goldstone AH, Boogaerts MA, Ferrant A, Demuynck HMS, Link H, Zander A, Barge A and Borkett K (1996) Randomised trial of filgrastim-mobilised peripheral blood progenitor cell transplantation versus autologous bone-marrow transplantation in lymphoma patients. *Lancet* **347**: 353–357

Sheridan WP, Morstyn G, Wolf M, Dodds A, Lusk J, Maher D, Layton JE, Green MD, Souza L and Fox RM (1989) Granulocyte colony-stimulating factor and neutrophil recovery after high dose chemotherapy and autologous bone marrow transplantation. *Lancet* **2**: 891–895

Sheridan W, Begley G, Juttner C, de Luca E, To LB, Szer J, Maher D, Watson D, Grigg A, Cebon J, Morstyn G, McGrath K, Green M, Tomita D, Hoffman E and Fox RM (1992) Effect of different doses and schedules of r-metHuG-CSF (Filgrastim) on mononuclear cell and PBPC collections and haematopoietic recovery after high dose chemotherapy (HDC) and infusion of r-metHuG-CSF mobilised peripheral progenitor cells (PBPC) without bone marrow. *Blood* **80**: 331a

Shimazaki C, Oku N, Uchiyama H, Yamagata N, Tatsumi T, Hirata T, Ashihara E, Okawa K, Goto H, Inaba T, Fujita N, Haruyama H and Nakagawa M (1994) Effect of granulocyte colony-stimulating factor on hematopoietic recovery after peripheral blood progenitor cell transplantation. *Bone Marrow Transplant* **13**: 271–275

Smith TJ, Hillner BE, Yanowich S, Sshmitz N, Lynch DC, Boogaerts M, Ferrant A, Link H and Zander A (1995) Economic analysis of peripheral blood progenitor cell (PBPC) or autologous bone marrow (ABM) transplant in relapsed Hodgkin's disease (HD) and non-Hodgkin's lymphoma (NHL). *Proc Am Soc Clin Oncol* **80**: 314

Spitzer G, Adkins DR, Spencer V, Dunphy FR, Petruska PJ, Velasquez WS, Bowers CE, Kronmueller N, Niemeyer R and McIntyre W (1994) Randomised study of growth factors post-peripheral-blood stem-cell transplant: neutrophil recovery is improved with modest clinical benefit. *J Clin Oncol* **12**: 661–670

Stahel RA, Jost LM, Cerny T, Pichert G, Honegger H, Tobler A, Jacky E, Fey M and Platzer E (1994) Randomised study of recombinant human granulocyte colony-stimulating factor after high-dose chemotherapy and autologous bone marrow transplantation for high-risk lymphoid malignancies. *J Clin Oncol* **12**: 1931–1938

The International Non-Hodgkin's lymphoma Prognostic Factors Project (1993) A predictive model for aggressive non-Hodgkin's lymphoma. *N Engl J Med* **329**: 987–994

Torres Gomez A, Jimenez MA, Alvarez MA, Rodriguez A, Martin C, Garcia MJ, Flores R, Sanchez J, de la Torre MJ and Herrera C (1995) Optimal timing of granulocyte colony-stimulating factor (G-CSF) after bone marrow transplantation. *Ann Hematol* **71**: 65–70

© Cancer Research Campaign 1998